# Placental mTOR signalling links mitochondrial dysfunction, nutrient transport and neonatal beta cell perturbations in mice

**DOI:** 10.1007/s00125-025-06542-z

**Published:** 2025-10-09

**Authors:** Megan Beetch, Eunice Oribamise, Seokwon Jo, Briana Clifton, Sarah Larson, Alex Hausmann, Alicia Wong, Brian Akhaphong, Elizabeth Morgan, Emilyn U. Alejandro

**Affiliations:** 1 Department of Integrative Biology and Physiology, University of Minnesota Medical School, Minneapolis, MN USA; 2 Stem Cell Institute, University of Minnesota Medical School, Minneapolis, MN USA; 3 UConn Health, University of Connecticut, Farmington, CT USA; 4 Department of Obstetrics, Gynecology and Women’s Health, University of Minnesota, Minneapolis, MN USA

**Keywords:** Amino acid transport, Beta cells, Insulin secretion, Mitochondria, mTOR signalling, Placenta

## Abstract

**Aims/hypothesis:**

Fetal programming of metabolic health is influenced by the *in utero* environment. The placental nutrient sensor mechanistic target of rapamycin (mTOR) is implicated in regulating fetal growth and programming of offspring metabolic health, but the mechanisms are unknown.

**Methods:**

Using a placental mTOR deficiency model to induce fetal growth restriction (FGR), we investigated mTOR-modulated placental mitochondrial function, nutrient transport and developmental programming of pancreatic beta cells, which are exquisitely sensitive to nutrient levels in utero.

**Results:**

We found defects in placental mitochondria function and morphology that were specific to placentas of mTOR knockout (mTORKO) mice. Despite smaller placentas and FGR in both sexes, nutrient transporter expression and leucine flux were paradoxically increased in female mTORKO placentas. Female fetuses exposed to placental mTOR deficiency (mTORKO^pl^) displayed significantly reduced circulating insulin without neonatal perturbations in insulin secretion. However, average beta cell size and proliferation were increased in mTORKO^pl^ female fetuses, possibly driven by system A (SNAT) amino acids, suggesting an immature beta cell phenotype. Adult mTORKO^pl^ female offspring exhibit increased susceptibility to diet-induced obesity, insulin resistance and inability to mount a beta cell mass response to a hypernutrient environment.

**Conclusions/interpretation:**

Our novel in vivo model of direct placental mTOR-driven FGR provides strong evidence linking placental dysfunction and amino acid transport to proper programming of beta cells in early life.

**Graphical Abstract:**

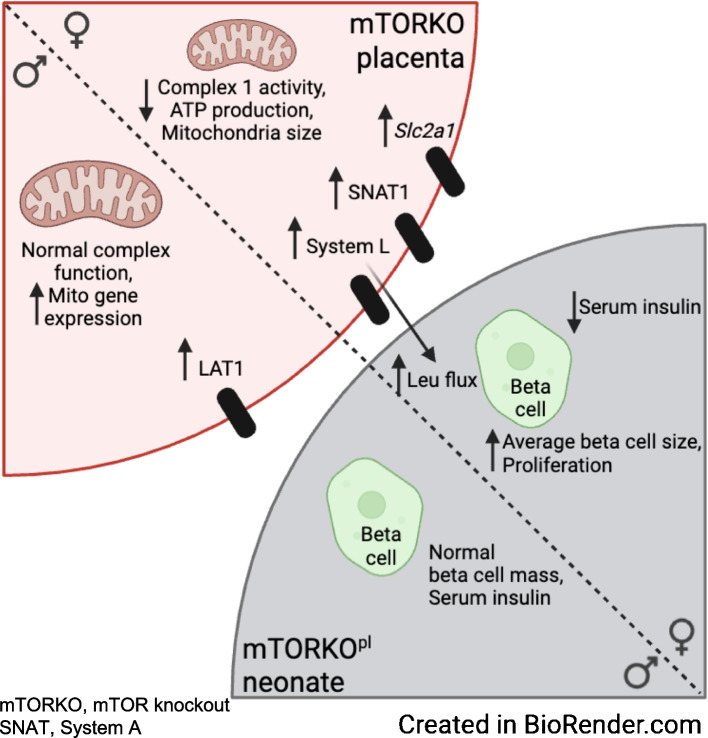

**Supplementary Information:**

The online version contains peer-reviewed but unedited supplementary material available at 10.1007/s00125-025-06542-z.


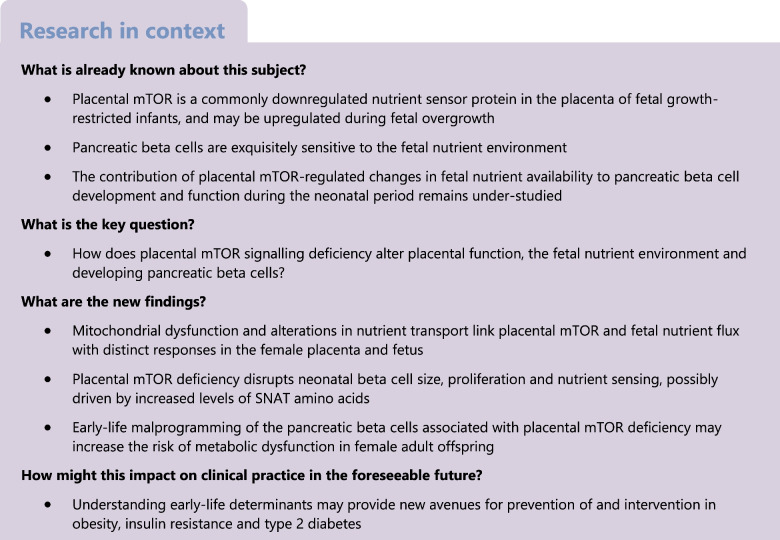


## Introduction

Decades of evidence link in utero factors with shaping risk for obesity and type 2 diabetes [1–3]. Fetal growth restriction (FGR) is a determinant of adverse long-term health. FGR occurs when a fetus fails to reach its genetic growth potential, often due to placental insufficiency [4]. Mechanistic target of rapamycin (mTOR) is a nutrient sensor kinase that is commonly downregulated in FGR placentas and upregulated during fetal overgrowth [5–7]. Our previous research demonstrated that placenta-specific mTOR deficiency significantly reduces fetal weight, mimicking an FGR phenotype [8]. Adult female offspring with reduced placental mTOR displayed exacerbated obesity and glucose metabolism dysfunction [8]. A strong correlation between placental mTOR status and offspring metabolic health trajectory has been established [4].

Placental mitochondrial dysfunction is implicated in pregnancy complications, including FGR [9]. Inhibition of mTOR by siRNA results in mitochondrial mass deficits and dysfunction in primary human trophoblasts [10]. The impact of placental mTOR deficiency on mitochondrial function in vivo remains undefined. Placental nutrient transport is a key factor in proper fetal growth. Ex vivo studies have shown that placental mTOR inhibition reduces amino acid transport, correlating with FGR [11–13]. Most amino acid uptake is modulated by amino acid transporter systems [12], including system L and system A. Glucose transport is another major determinant of adequate growth [13, 14]. Positive associations between placental mTOR signalling, nutrient transporters, and fetal growth have been reported [6, 7, 15]. However, the consequences of direct placental mTOR manipulation on nutrient transport in vivo have not been elucidated.

Nutrient perturbations impair neonatal beta cell mass and glucose tolerance in adulthood [2, 3, 16, 17]. Suboptimal nutrient availability can impact critical beta cell proteins [18, 19] and beta cells are particularly sensitive to nutrient flux early in life [20]. Therefore, proper neonatal beta cell function may determine long-term responses to various nutrient environments. In the present study, we aimed to determine whether placental mTOR deficiency in vivo alters mitochondrial function and nutrient transport, and to test the hypothesis that placental mTOR deficiency perturbs nutrient flux to the fetus and impacts neonatal beta cell mass and function, negatively influencing long-term metabolic health.

## Methods

### Mouse model

A mouse model with loss of placental mTOR signalling was generated as previously reported [8]. Cyp19-cre recombinase [21] was used with loxP-flanked sites in the *Mtor* gene (Jackson Laboratory, strain #011009). Each litter comprises Cyp19-cre-positive and Cyp19-cre-negative offspring, used as littermate controls. Details are provided in the electronic supplementary materials (ESM Methods). Animal studies were performed in accordance with the University of Minnesota Institutional Animal Care and Use Committee (IACUC: protocol 2106–39213). Our study examined male and female placentas/neonates and found sex differences in the parameters measured; sex was therefore included as a biological variable in our analyses.

### Embryonic tissue collection and processing

The presence of a copulation plug indicates embryonic day 0.5 (e0.5). Placentas containing maternal decidua and embryonic pancreas were collected for RNA and protein extraction or paraffin embedding. Paraffin-embedded tissues were sectioned at a thickness of 5 µm through the entirety of the pancreas.

### BeWo cells and rapamycin treatment

Human placental choriocarcinoma BeWo cells were gifted by S. Wernimont (University of Minnesota), authenticated, and checked for mycoplasma contamination. Culture conditions are given in ESM Methods. Rapamycin treatment at 30 or 100 nmol/l concentration was applied for 24 h before collection.

### Human placenta collection and processing

Placenta tissue samples were collected with the oversight of the University of Minnesota centralised biorepository and bioregistry for patient consent (BioNet) and under the approval of the University of Minnesota Institutional Review Board (IRB number STUDY00011993). Clinical characteristics and collection/processing details are given in ESM Table 1.

### Mouse trophoblast isolation and bulk RNA sequencing

Mouse placenta tissues were digested using HBSS+ cations, 30 μg/ml DNase 1, 2.5% trypsin, 1 mg/ml collagenase, at 37°C, followed by filtering through a cell strainer, erythrocyte lysis and resuspension of cells in FACS buffer. Cells were submitted to the University of Minnesota Flow Cytometry Resource for GFP+ cell sorting, and collected for RNA isolation using RNeasy Plus Micro kits (Qiagen). Bulk RNA sequencing procedures and analysis details are provided in ESM Methods.

### Quantitative RT-PCR

RNA samples were prepared using TRIzol with chloroform and quantified using a Nanodrop spectrophotometer (Thermo Scientific). A high-capacity cDNA reverse transcription kit (Applied Biosystems) was used for cDNA conversion. PCR was performed using the Applied Biosystems Q6 machine and SYBR Green reagent. β-Actin was used as the reference gene. Primer sequences are given in ESM Table 2.

### Western blotting

Placentas or BeWo cells were lysed by homogenisation in RIPA buffer containing 1% SDS and protease and phosphatase inhibitors. Following BCA assay, 35 μg protein placental lysate or 50 μg BeWo cell lysate was resolved by SDS–PAGE, transferred to PVDF membrane, blocked with 5% non-fat dry milk, and incubated with primary antibodies overnight, prior to treatment with HRP-conjugated secondary antibodies. The primary antibodies are listed in ESM Methods. Blots were visualised using SuperSignal West Pico PLUS (Thermo Scientific), according to the manufacturer’s instructions. Densitometry analysis was performed using Fiji ImageJ software (https://imagej.net/software/fiji/downloads).

### Seahorse and mitochondrial mass

#### Frozen mouse placenta

A previously published protocol for mitochondria isolation and Seahorse analysis from previously frozen tissues was followed [22]. Sample and drug preparation details are given in ESM Methods. Mitochondrial respiration was measured using a Seahorse XFe96 Extracellular Flux analyser (Agilent Technologies).

#### Primary mouse trophoblasts and BeWo cell line

Primary mouse trophoblasts were isolated using a previously published method [23]. Briefly, mouse placentas were digested, and cell types were separated by Percoll gradient and centrifugation steps. Trophoblasts isolated from mTOR knockout (MTORKO) and control placentas were plated onto Seahorse XF96 plates for 48 h. BeWo cells were plated into Seahorse XFe96 plates and treated with 30 or 100 nmol/l rapamycin or DMSO vehicle for 24 h. Following treatment, the medium was switched to Seahorse assay medium. Mitochondrial respiration was measured using the Seahorse XF Cell Mito Stress Test kit for the Seahorse XFe96 Extracellular Flux analyser (Agilent Technologies). Oxygen consumption rate measurements were normalised to DNA concentration using a Quant-iT PicoGreen dsDNA kit.

To measure mitochondrial mass, total DNA was isolated from placentas using a QIAamp DNA Micro kit. Quantitative RT-PCR using primers flanking the mouse nuclear gene β2-microglobulin or mouse mitochondrial DNA (mtDNA) sequence was used to measure relative expression [24].

### Transmission electron microscopy

Mouse placenta biopsy punchouts were collected and fixed, and then sectioned using a diamond knife on a Leica Ultracut UCT microtome at a thickness of 70–100μm, and stained using 3% aqueous uranyl acetate and Sato’s lead citrate stain. Grids were imaged on a Philips CM12 transmission electron microscope at 60 kV at the University of Minnesota Imaging Center. Images were captured throughout the placenta at 3000× and 10,000× magnification to clearly identify nuclei, cell boundaries and mitochondria. Manual counting of mitochondria and size binning using Fiji ImageJ software were performed in *n*=5–8 cells per placenta.

### Immunostaining

Paraffin-embedded placentas were sectioned and immunostained using a rabbit-specific HRP/DAB (ABC) Detection IHC kit (Abcam), according to the manufacturer’s instructions. Primary antibodies with corresponding dilution factors are listed in ESM Table 3. Analysis details are given in ESM Methods.

Paraffin-embedded pancreas sections were immunostained as previously described [8, 25]. Primary antibodies with dilution factors are given in ESM Table 3. Visualisation and imaging were performed using a Keyence fluorescence microscope at the indicated magnifications. Analysis of beta and alpha cell mass was performed as previously described [25]. Details are provided in ESM Methods.

### Leucine and glucose flux

Dams were catheterised via the jugular vein. For measurement of leucine flux, 3,700,000 Bq ^3^H-leucine in physiological saline (154 mmol/l NaCl) was administered under isoflurane anaesthesia for 10 min [8]. For measurement of glucose flux, 3,700,000 Bq non-metabolisable ^3^H-methyl-d-glucose in physiological saline was administered under isoflurane anaesthesia for 3 min [26, 27]. Dams were killed by cervical dislocation. Prior to injection, and 3 and 10 min after glucose or leucine injection, respectively, maternal serum was collected to confirm ^3^H circulation. Placentas and fetuses were rapidly harvested, digested in Biosol, and then Bioscint was added (both National Diagnostics). Digested fetal tissue was diluted 1:5 for accurate measurement on the TriCarb liquid scintillation counter (Perkin Elmer). Disintegrations per minute were measured, and used to calculate leucine or glucose transfer relative to placental weight or fetal weight. Fold change was calculated relative to same-sex littermate control animals in each litter (*n*=6 dams for leucine and *n*=3 dams for glucose). Outliers were identified on the basis of being outside the upper and lower limits of the IQR.

### Glucose- and leucine-stimulated insulin secretion

Islets were isolated from 7-day-old neonates by collagenase digestion. Following overnight rest, islets were prepared as previously described [28, 29]. Details for static and dynamic glucose-stimulated insulin secretion (GSIS) conditions are given in ESM Methods. Dynamic leucine-stimulated insulin secretion (LSIS) was also measured in combination with glucose conditions. Insulin concentrations were determined using Ultrasensitive Mouse Insulin ELISA kits (ALPCO) and normalised by DNA determined using a PicoGreen dsDNA assay.

### Amino acid treatment of embryonic pancreas explants

Pancreases were harvested from wild-type fetuses at e17.5 and cultured for 72 h in control, branched chain amino acid (BCAA) or system A amino acid (SNAT AA) medium. Details for the culture media and conditions are given in ESM Methods. Media were changed every 24 h. After 72 h incubation with control medium, BCAA medium or SNAT AA medium, explants were fixed, processed and embedded. Sectioning, staining and analyses were executed as described above and in ESM Methods.

### Statistical analysis

Data are presented as mean ± SEM. Placental weights, fetal weights and placental efficiency were analysed using two-way ANOVA, with sex and genotypes as factors, and by Sidak’s multiple comparisons test. Expression data were analysed using an unpaired, two-tailed *t* test or one-way ANOVA with Tukey post hoc test. Beta cell, alpha cell and other staining were analysed using an unpaired, two-tailed *t* test. Static GSIS experiments were analysed using two-way ANOVA with Sidak’s multiple comparisons test or an unpaired, two-tailed *t* test. Dynamic GSIS/LSIS experiments were analysed using the area under curve followed by unpaired, two-tailed *t* test. Analyses and data visualisation were performed in GraphPad PRISM version 8. The significance threshold was *p*<0.05.

## Results

### Placental mTOR deficiency causes FGR in mice

**Fig. 1:**
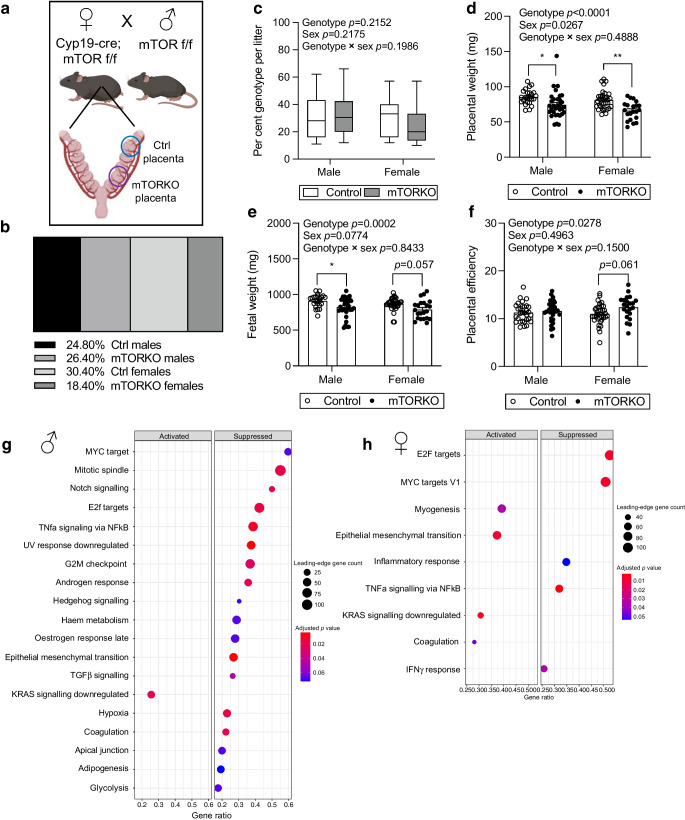
Placental mTOR deficiency results in FGR. (**a**) Breeding scheme and possible placental genotypes within a litter. (**b**) Sex and genotype proportions in e17.5 litters. (**c**) Proportion of each genotype by sex per litter. (**d**) Weight of control and mTORKO male and female placentas (*n*=21–35). (**e**) Weight of control and mTORKO^pl^ male and female fetuses (*n*=22–34). (**f**) Placental efficiency for control and mTORKO^pl^ male and female offspring determined by fetal weight divided by placental weight (*n*=22–35). (**g**, **h**) Dot plots from gene set enrichment analyses (GSEA) comparing trophoblasts from (**g**) male and (**h**) female control and mTORKO placentas. Statistical analyses were performed using two-way ANOVA, with sex and genotype as factors. Asterisks indicate significance: *p<0.05, **p<0.01. Ctrl, control

Our breeding scheme is shown in Fig. 1a. Placental sex and genotype proportions indicate that mTORKO female fetuses (18.40%) are present at below the expected proportion of 25% (Fig. 1b). When analysed per litter, the proportion of mTORKO female fetuses was not statistically lower (Fig. 1c). mTORKO caused a significant reduction in placental weight during mid-gestation in both male and female placentas (ESM Fig. 1a), with no differences in fetal weight at this timepoint (ESM Fig. 1b). Placental efficiency was not different during mid-gestation (ESM Fig. 1c). In late gestation, placental mTOR deficiency resulted in significantly reduced placental weight (Fig. 1d and ESM Fig. 1d) and FGR in both male and female fetuses (Fig. 1e and ESM Fig. 1e). Placental mTOR deficiency increased placental efficiency with no differences between sexes (Fig. 1f).

Reduction of placental mTOR was confirmed in mTORKO placentas (ESM Fig. 1f; genotype effect *p*=0.0004, genotype × sex interaction *p*=0.0774). The degree of mTOR inhibition was comparable in male and female mTORKO placentas (ESM Fig. 1f). Notably, mTOR was not statistically significantly reduced in the male placentas when analysed using multiple comparisons (*p*=0.0983), whereas female placentas displayed a significant reduction (ESM Fig. 1f). Bulk RNA sequencing of isolated trophoblasts from control and mTORKO placentas revealed that mTOR mediation of cell signalling pathways was distinct in each sex. In male mTORKO placentas, Notch signalling, TGFβ signalling, hormone responses and hypoxia were significantly decreased (Fig. 1g). In female mTORKO placentas, cell replication and inflammation pathways were significantly decreased, while epithelial mesenchymal transition signalling was increased (Fig. 1h). TNFα signalling by NF-κB was suppressed and Kirsten rat sarcoma viral oncogene homologue (KRAS) signalling was increased in both male and female mTORKO placentas (Fig. 1g, h). These findings highlight the myriad disruptions induced by mTOR deficiency in the placenta.

### Mitochondrial dysfunction and suboptimal ultrastructure in the mTORKO placenta

**Fig. 2:**
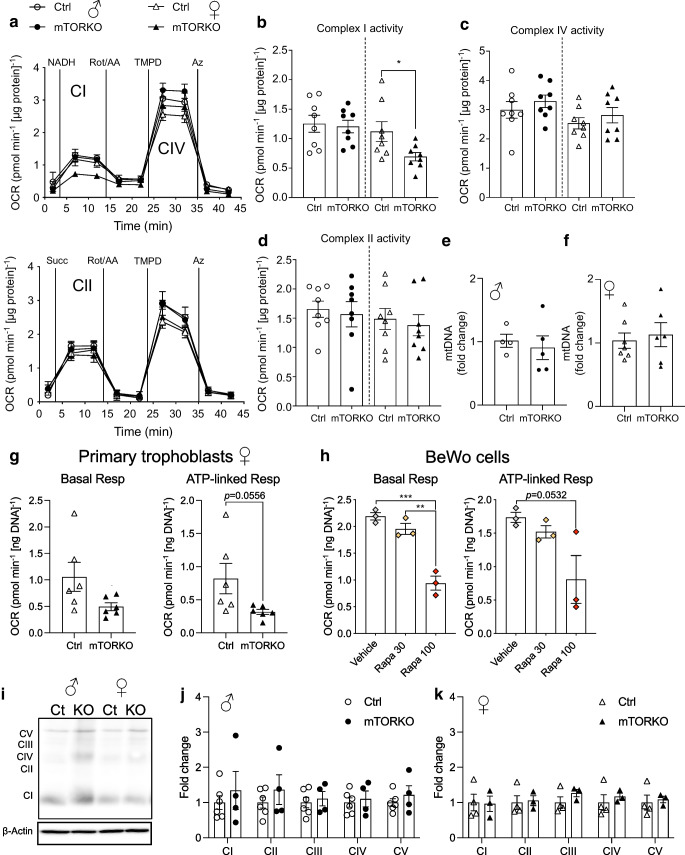
Mitochondrial function in the mTOR-deficient placenta. (**a**) Seahorse traces, with quantification of (**b**) complex I activity, (**c**) complex IV activity, and (**d**) complex II activity in control and mTORKO male and female placentas (*n*=7–8). Circles, male; triangles, female. (**e**, **f**) Mitochondrial mass quantified by mtDNA in (**e**) male and (**f**) female placentas of control and mTORKO mice (*n*=4–7). (**g**) Quantification of basal respiration and ATP-linked respiration in primary trophoblasts isolated from female control and mTORKO placentas (*n*=6). (**h**) Quantification of basal respiration and ATP-linked respiration in BeWo cells in response to treatment with 30 or 100 nmol/l rapamycin (Rapa 30/Rapa 100) (*n*=3). (**i**–**k**) Western blot (**i**) and quantification of total oxidative phosphorylation complexes in (**j**) male and (**k**) female placentas of control and mTORKO mice (*n*=3–6). Statistical analyses were performed using an unpaired two-tailed *t* test or one-way ANOVA with Tukey’s post hoc test. Asterisks indicate significance: *p<0.05, **p<0.01, ***p<0.001. AA, amino acid; Az, azide; CI, complex I; CII, complex II; CIV, complex IV; Ct or Ctrl, control; OCR, oxygen consumption rate; Resp, respiration; Rot, rotenone; Succ, succinate; TMPD, tetramethylphenylenediamine

Mitochondrial dysfunction is linked to FGR because mitochondrial capacity is necessary for proper placental growth and function, including nutrient transport to the growing fetus. Mitochondrial function was first measured by Seahorse assay using whole frozen placenta tissue, which allows assessment of individual oxidative phosphorylation complex activity. mTOR deficiency reduced complex I activity in the female placentas (Fig. 2a, b and ESM Fig. 2a), with no significant impact on complex II or complex IV (Fig. 2a–d and ESM Fig. 2b, c). Oxidative phosphorylation complex activity (Fig. 2a–d and ESM Fig. 2a) and mtDNA content (Fig. 2e) were comparable in male control and mTORKO placentas. Female mTORKO placentas showed no differences in mtDNA content compared with female controls (Fig. 2f).

To complement our findings in frozen tissue, we assessed mitochondrial function in primary mouse trophoblasts isolated from control and mTORKO placentas. This method allows assessment of the live cell oxygen consumption rate. We found decreases in basal and ATP-linked respiration in female mTORKO trophoblasts, but these did not reach statistical significance (*p*=0.0752 and *p*=0.0556, respectively) (Fig. 2g). No differences in basal or ATP-linked respiration were detected in male mTORKO trophoblasts (ESM Fig. 2d). We validated the mTOR–mitochondria relationship using the BeWo cell line. Treatment with 30 or 100 nmol/l rapamycin for 24 h reduced mTOR signalling to 37% and 22% of control levels, respectively (ESM Fig. 2e, f). mtDNA content was not statistically different in 30 nmol/l rapamycin-treated cells (*p*=0.072) or 100 nmol/l rapamycin-treated cells (*p*=0.09) compared with control (ESM Fig. 2g). The live cell oxygen consumption rate was significantly blunted in BeWo cells treated with 100nmol/l rapamycin (ESM Fig. 2h). As in mTORKO primary trophoblasts, deficits in basal and ATP-linked respiration (Fig. 2h) were detected in BeWo cells with inhibited mTOR signalling. Maximum respiration, proton leak, space capacity and non-mitochondrial respiration were unchanged (ESM Fig. 2i–l). These live cell data suggest that mTOR inhibition in placental cells results in reduced ATP production.

Protein expression of oxidative phosphorylation complexes I–V was not different by genotype in either sex (Fig. 2i–k). However, gene expression of regulators of mitochondrial genome transcription (*Tfam*), dynamics (*Mfn1* and *Mfn2*), oxidative stress (*Nrf2*) and energy metabolism (*Pparg*) was increased in male mTORKO placentas, but not female mTORKO placentas (ESM Fig. 2m, n).

**Fig. 3:**
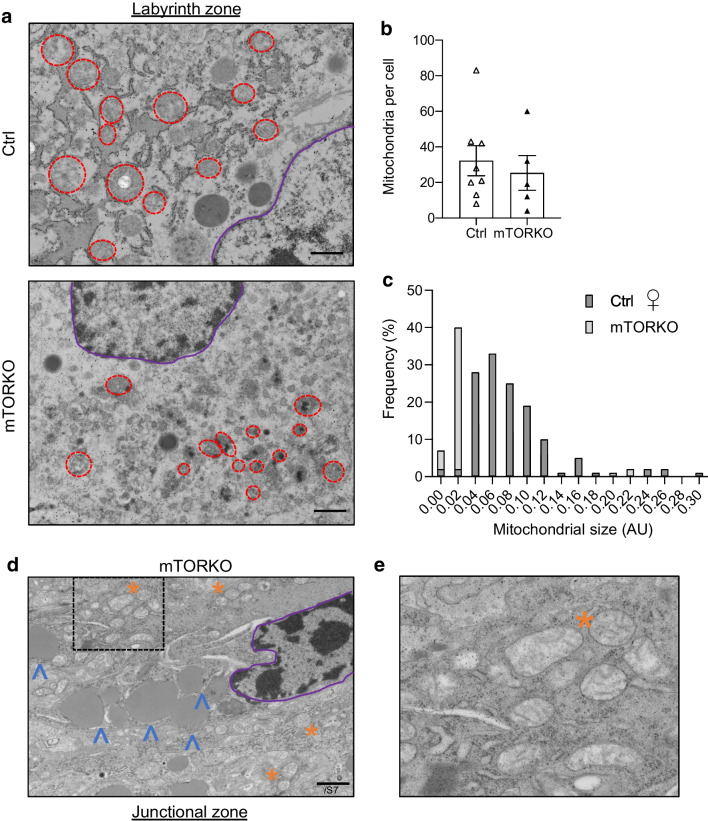
Mitochondrial morphology in the mTOR-deficient placenta. (**a**) Representative ×10,000 images of the labyrinth zone of control and mTORKO female placentas. Representative mitochondria are circled in red; the nucleus is outlined in purple. (**b**) Quantification of mitochondria per cell (*n*=5–8). (**c**) Frequency of each size of mitochondria in control and mTORKO female placentas. AU, arbitrary units. (**d**) ×10,000 images of the junctional zone in a female mTORKO placenta. Electron-transparent mitochondria are indicated by orange asterisks, with a magnified image in (**e**); lipid accumulation is indicated by blue arrowheads. Scale bar, 1 μm. Statistical analyses were performed using an unpaired two-tailed *t* test with significance set at *p*<0.05 (no significant findings in the figure). Ctrl, control

Transmission electron microscopy allows observation of subcellular ultrastructure, including mitochondria. In the labyrinth zone, we observed comparable mitochondria counts per cell in female control and mTORKO placentas (Fig. 3a, b). However, there were considerably more small mitochondria in the female mTORKO placentas (Fig. 3c). In the junctional zone of the female mTORKO placenta, the mitochondria appear to be electron-transparent (Fig. 3d, e), which may suggest unhealthy mitochondria and electron leak [30]. Accumulation of lipids was observed in the female mTORKO placentas (Fig. 3d). Together, these findings indicate disruptions of mitochondrial morphology and nutrient storage perturbations in the female mTORKO placentas.

### Glucose transporter expression and flux in the mTORKO placenta

To understand alterations in nutrient transport in mTORKO placentas, glucose transporter expression and flux were measured. There was an increase in *Slc2a1* (GLUT1) in male mTORKO placentas (*p*=0.0554) and female mTORKO placentas (ESM Fig. 3a, b). We measured unidirectional flux using ^3^H-labelled glucose. We found no differences by genotype in terms of glucose detected in the male or female fetuses (ESM Fig. 3c, e) or placentas (ESM Fig. 3d, e). We noted significantly increased glycogen cell numbers in the junctional zone of the mTORKO male placentas (ESM Fig. 3f, g) and female placentas (ESM Fig. 3h, i).

### Elevated system A expression in the mTORKO placenta

**Fig. 4:**
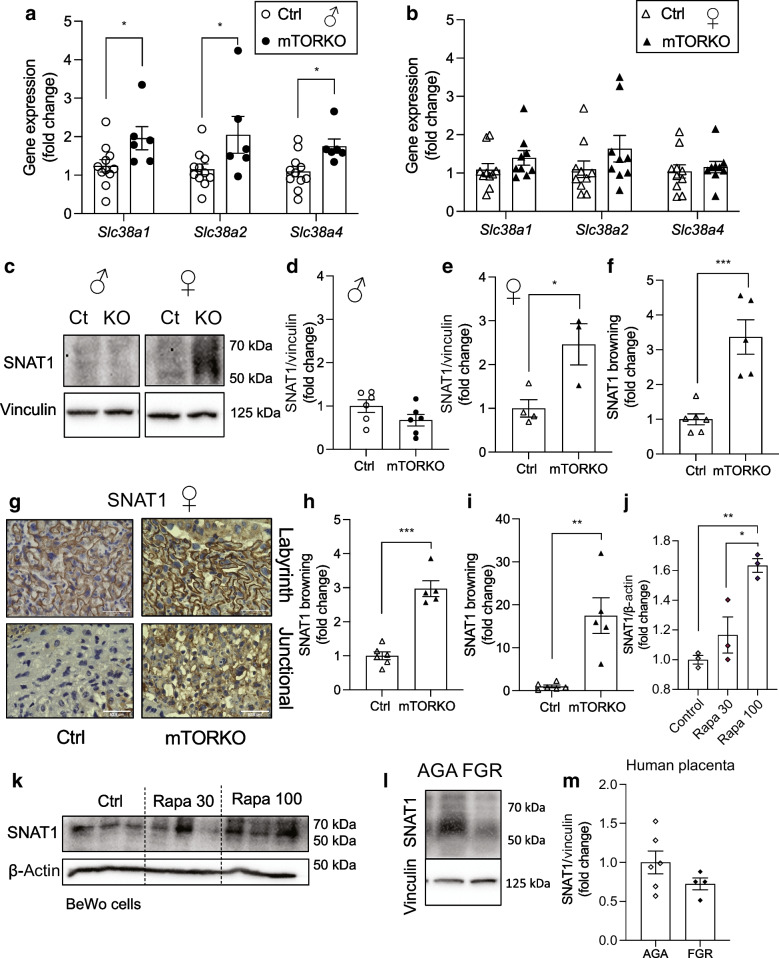
Elevated expression in late gestation. (**a**) Expression of *Slc38a1* (SNAT1), *Slc38a2* (SNAT2) and *Slc38a4* (SNAT4) in male mTORKO placentas and littermate control placentas (*n*=6–11). (**b**) Expression of *Slc38a1* (SNAT1), *Slc38a2* (SNAT2) and *Slc38a4* (SNAT4) in female mTORKO placentas and littermate control placentas (*n*=9–10). (**c**) Western blot for SNAT1 expression in mTORKO placentas. Vinculin is used as a loading control. (**d**, **e**) Quantification of the SNAT1 blot for (**d**) male mTORKO placentas compared with littermate control placentas (*n*=6) and (**e**) female mTORKO placentas compared with littermate control placentas (*n*=3–4). (**f**) Quantification of whole-placenta SNAT1 immunohistochemistry staining for female mTORKO placentas and littermate controls (*n*=5–6). (**g**) ×60 images of SNAT1 immunohistochemistry staining by zone in female control and mTORKO placentas. Scale bar, 52.8 μm. (**h**, **i**) Quantification for (**h**) the labyrinth zone and (**i**) the junctional zone (*n*=5–6); normalised to female control placentas using fold-change analysis. (**j**, **k**) Quantification (**j**) and images (**k**) of a western blot measuring SNAT1 expression in BeWo cells treated with 30 or 100 nmol/l rapamycin (Rapa 30/Rapa 100) (*n*=3). (**l**, **m**) Images (**l**) and quantification (**m**) of a western blot measuring SNAT1 expression in human AGA and FGR placentas (*n*=4–6). Statistical analyses were performed using an unpaired two-tailed *t* test or one-way ANOVA with Tukey’s post hoc test. Asterisks indicate significance: *p<0.05, **p<0.01, ***p<0.001. Browning indicates the intensity of 3,3′-diaminobenzidine (DAB) staining. Ct or Ctrl, control

We measured the expression of amino acid transporters in the placenta. Expression of *Slc38a1* (encoding SNAT1), *Slc38a2* (SNAT2) and *Slc38a4 (*SNAT4) was significantly increased in the male mTORKO placentas compared with control placentas (Fig. 4a). We found no differences in *Slc38a* gene expression when comparing female control and mTORKO placentas (Fig. 4b). SNAT1 has been implicated as responsible for system A activity later in gestation [31]. To our surprise, we did not detect significant differences in SNAT1 protein levels in the male placentas by western blotting (Fig. 4c, d) or immunohistochemistry (ESM Fig. 4a–c). However, we did observe increased SNAT1 protein levels in the whole female mTORKO placentas by western blotting (Fig. 4c, e) and immunohistochemistry (Fig. 4f), with robust and significant increases in the labyrinth zone (Fig. 4g, h) and junctional zone (Fig. 4g, i). BeWo cells treated with rapamycin showed increased SNAT1 protein expression in a dose-dependent manner (Fig. 4j, k). Due to the link between placental mTOR, amino acid transporter expression and birthweight in humans [4, 32], SNAT1 expression was also measured in term human placentas from appropriate-for-gestational age (AGA) and FGR newborns. SNAT1 protein levels in the AGA and FGR placentas were comparable (Fig. 4l, m). Together, our data suggest that direct mTOR deletion in vivo or in vitro increases SNAT1 expression.

### Increased system L expression and flux in the mTORKO placenta

**Fig. 5:**
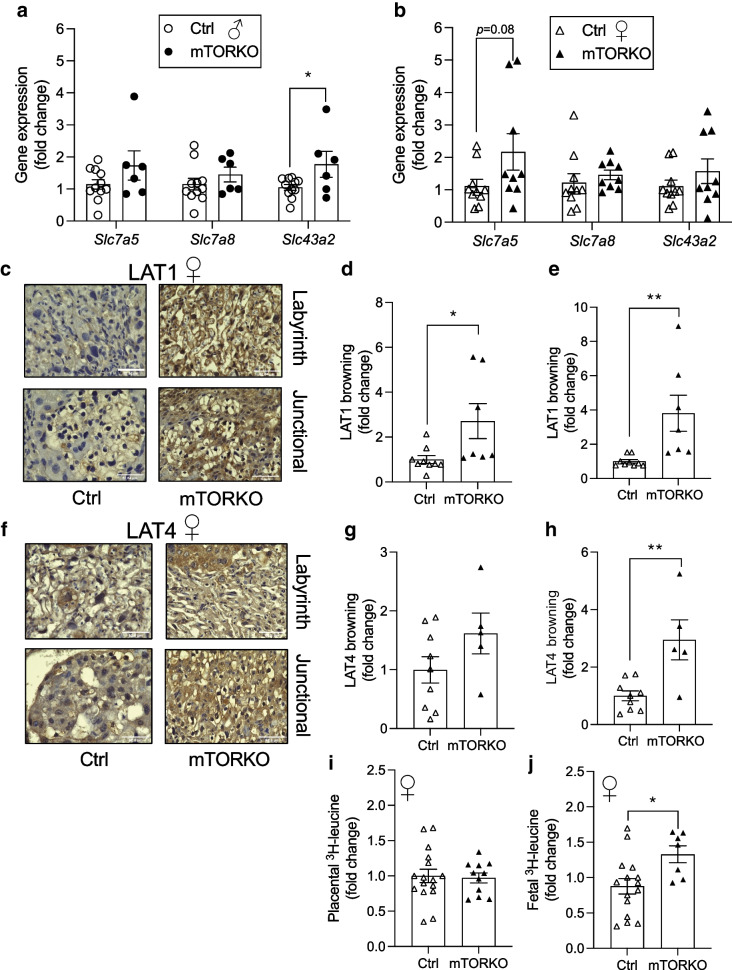
Increased expression of system L subunits in late gestation. (**a**) Expression of *Slc7a5* (LAT1), *Slc7a8* (LAT2) and *Slc43a2* (LAT4) in male mTORKO placentas and littermate control placentas (*n*=6–11). (**b**) Expression of *Slc7a5* (LAT1), *Slc7a8* (LAT2) and *Slc43a2* (LAT4) in female mTORKO placentas and littermate control placentas (*n*=9–10). (**c**) ×60 images of LAT1 immunohistochemistry staining by zone in female control and mTORKO placentas. Scale bar, 52.8 μm. (**d**, **e**) Quantification for (**d**) the labyrinth zone and (**e**) the junctional zone (*n*=7–9) normalised to female control placenta using fold-change analysis. (**f**) ×60 images of LAT4 immunohistochemistry staining by zone in female control and mTORKO placentas. Scale bar, 52.8 μm. Quantification for (**g**) the labyrinth zone and (**h**) the junctional zone (*n*=5–9) normalised to female control placenta using fold-change analysis. (**i**, **j**) ^3^H-leucine detected in female placentas (*n*=11–17) (**i**) and fetuses (*n*=7–15) (**j**) following administration to maternal circulation. Statistical analyses were performed using an unpaired two-tailed *t* test. Asterisks indicate significance: *p<0.05, **p<0.01. Browning indicates the intensity of 3,3′-diaminobenzidine (DAB) staining. Ctrl, control

**Fig. 6:**
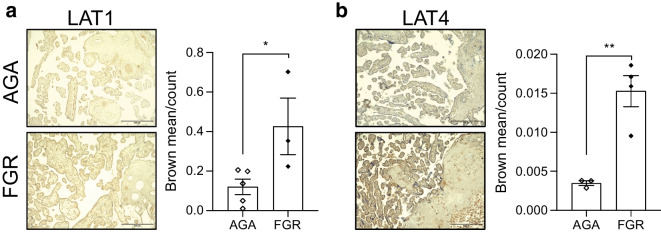
Increased expression of system L subunits in human FGR. (**a**) ×10 images and quantification of LAT1 immunohistochemistry staining in human AGA and FGR placentas (*n*=3–5). (**b**) ×10 images and quantification of LAT4 immunohistochemistry staining in human AGA and FGR placentas (*n*=3–4). Scale bar, 500 μm. Browning indicates the intensity of 3,3′-diaminobenzidine (DAB) staining. Statistical analyses were performed using an unpaired two-tailed *t* test. Asterisks indicate significance: *p<0.05, **p<0.01

In our in vivo model of direct placental mTOR deletion, we only found differences in expression of *Slc43a2* (encoding LAT4) in male mTORKO placentas compared with control placentas (Fig. 5a), and no significant gene expression differences in female mTORKO placentas (Fig. 5b). However, we found significant increases in LAT1 protein expression in both the labyrinth and junctional zones of mTORKO female placentas compared with control placentas (Fig. 5c–e). A significant increase in LAT4 protein expression in the junctional zone of female mTORKO placentas was also detected (Fig. 5f–h). Interestingly, we also detected increased LAT1 protein expression in the junctional zone of male mTORKO placentas (ESM Fig. 4d–f), but no significant differences in LAT4 protein expression between male mTORKO placentas and control placentas (ESM Fig. 4g–i). We used ^3^H-labelled leucine to assess whether increased system L transporter expression led to increased flux. We found no differences in ^3^H-leucine levels in the female placentas by genotype (Fig. 5i). However, we detected significantly increased ^3^H-leucine in female fetuses exposed to placental mTORKO (Fig. 5j and ESM Fig. 4j), suggesting increased flux. In contrast, we previously reported that ^3^H-leucine flux is reduced in male fetuses exposed to placental mTORKO [8]. System L subunit expression was also measured in the placentas of FGR-complicated pregnancies. As for mTORKO mouse placentas, we detected increased LAT1 and LAT4 protein in FGR human placentas (Fig. 6a, b). Our findings suggest an increase in system L expression and flux in the female mTORKO placenta that is sufficient to increase leucine transport to the fetus, which may be relevant in human FGR.

### Beta cell function in neonatal offspring exposed to placental mTOR deficiency

**Fig. 7:**
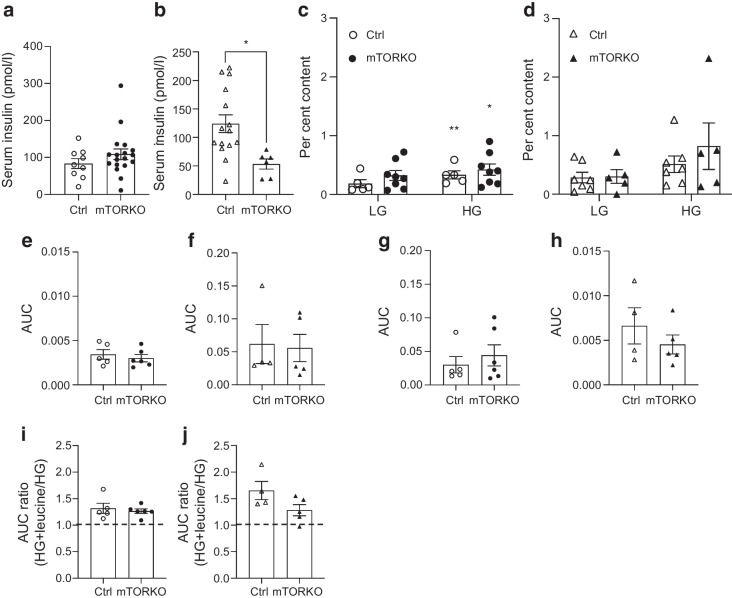
Neonatal GSIS differences in male and female mTORKO^pl^ offspring. (**a**, **b**) Serum insulin levels in late-gestation (e17.5) control and mTORKO^pl^ male fetuses (*n*=9–18) (**a**) and female fetuses (*n*=6–15) (**b**). (**c**, **d**) Static GSIS in response to low (LG, 2 mmol/l) and high (HG, 16.7 mmol/l) glucose concentrations in control and mTORKO^pl^ male neonates (*n*=5–8) (**c**) and control and mTORKO^pl^ female neonates (*n*=4–6) (**d**) presented as % insulin content. (**e**, **f**) Dynamic GSIS in response to low glucose (LG, 2 mmol/l) supplemented with l-leucine (1 mmol/l) in control and mTORKO^pl^ male (**e**) and female (**f**) neonates, presented as the AUC. (**g**, **h**) Dynamic GSIS in response to high glucose (HG, 16.7 mmol/l) in control and mTORKO^pl^ male (**g**) and female (**h**) neonates, presented as the AUC. (**i**, **j**) Dynamic GSIS in response to HG supplemented with leucine (1 mmol/l) in control and mTORKO^pl^ male (**i**) and female (**j**) neonates, presented as the AUC stimulation ratio. Statistical analyses were performed using an unpaired two-tailed *t* test or two-way ANOVA with Sidak’s multiple comparisons test. Asterisks indicate significance: *p<0.05, **p<0.01. Ctrl, control

We hypothesised that neonatal beta cell function may be perturbed in response to placental mTOR-mediated disruptions to the fetal nutrient flux. Indeed, we found significantly lower circulating insulin levels in placental mTORKO (mTORKO^pl^) female fetuses, but not in mTORKO^pl^ male fetuses (Fig. 7a, b). We did not detect any differences in circulating fetal glucagon (ESM Fig. 5a). These data suggest a functional defect in insulin secretion or clearance in female mTORKO^pl^ offspring.

Static GSIS experiments assessed islet responses to low glucose (2 mmol/l, LG) and high glucose (16.7mmol/l, HG), with no differences in insulin secretion by genotype being observed (Fig. 7c, d). Dynamic GSIS experiments assessed insulin secretion in response to glucose and leucine at higher resolution. In neonates there is a critical transition from amino acids to glucose as the main stimulus of insulin secretion [33]. We aimed to determine whether this transition was disrupted in mTORKO^pl^ offspring. Across sexes and genotypes, basal insulin secretion in response to LG (2 mmol/l) was not different (ESM Fig. 5c, d), nor was insulin secretion in response to LG + leucine (Fig. 7e, f and ESM Fig. 5e), high glucose only (HG, 16.7mmol/l) (Fig. 7g, h and ESM Fig. 5f) or HG + leucine (Fig. 7i, j, ESM Fig. 5f) in male or female offspring. Islet insulin content was comparable between mTORKO^pl^ offspring and their respective littermate controls (ESM Fig. 5g, h).

### Increased average beta cell size and proliferation in neonatal female mice exposed to placental mTOR deficiency

**Fig. 8:**
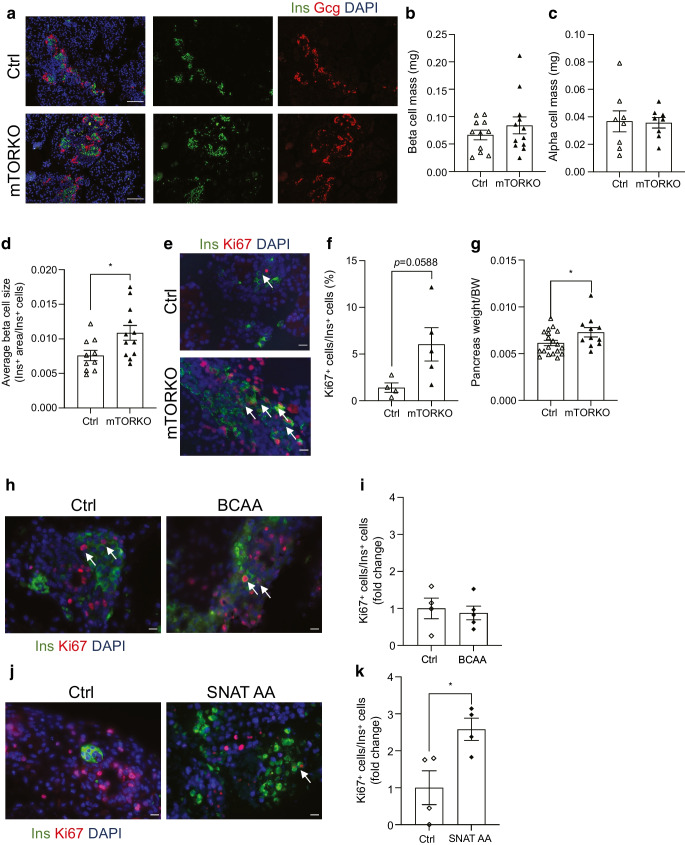
Increased average beta cell size and proliferation in mTORKO^pl^ female fetuses. (**a**) Representative images of islets stained for insulin (Ins; green) and glucagon (Gcg; red) from female littermate control and mTORKO^pl^ fetuses (e17.5). Scale bar, 50 μm. (**b**, **c**) Quantification of beta cell mass (*n*=11–12) (**b**) and alpha cell mass (*n*=8) (**c**). (**d**) Average beta cell size (*n*=11–12). (**e**) Representative ×60 images of the Ki67 proliferation marker in insulin-positive cells in female littermate control and mTORKO^pl^ fetuses (e17.5). Scale bar, 50 μm. Arrows indicate representative proliferating insulin-positive cells included in analyses. (**f**) Quantification of Ki67 in insulin-positive cells (*n*=4–5). (**g**) Pancreas weight normalised by body weight (BW) in female littermate control and mTORKO^pl^ e17.5 fetuses (*n*=11–19). (**h**, **i**) Representative ×60 images (**h**) and quantification (**i**) of Ki67 proliferation of insulin-positive cells in wild-type pancreas explants incubated with BCAA medium or control medium for 72 h. Scale bar. 50 μm. (**j**, **k**) Representative ×60 images (**j**) and quantification (**k**) of Ki67 proliferation of insulin-positive cells in wild-type pancreas explants incubated with SNAT AAs or control medium for 72 h. Scale bar. 50 μm. (**h**, **j**) Arrows indicate insulin-positive beta cells. Statistical analyses were performed using an unpaired two-tailed *t* test. Asterisks indicate significance: *p<0.05. Ctrl, control

To further investigate neonatal beta cells, we determined whether placental mTOR deficiency altered beta cell mass in the developing fetus. We found no differences in beta cell mass or alpha cell mass in male mTORKO^pl^ fetuses (ESM Fig. 6a–c) or mTORKO^pl^ female fetuses (Fig. 8a–c) compared with control fetuses. However, female mTORKO^pl^ fetuses had significantly increased average beta cell size (Fig. 8d) and an increase in proliferation in insulin-positive cells that approached significance (*p*=0.0588, Fig. 8e, f). Pancreas weight was also increased in the female mTORKO^pl^ fetuses (Fig. 8g).

We next assessed whether increasing BCAAs (leucine, isoleucine, valine), which are transported through system L, or amino acids that are transported through SNAT AAs (glutamine, alanine, serine) was sufficient to increase beta cell proliferation during late gestation (e17.5). After 3 days of culture with BCAAs, the embryonic pancreas did not display increased proliferation in insulin-positive cells (Fig. 8h, i). Leu, a BCAA and a potent activator of mTOR signalling, has been shown to reduce beta cell differentiation, while mTORC1, through genetic deletion, is reported to be required for pancreatic progenitor development [34, 35]. We did not observe any differences in beta cell mTOR signalling measured by phosphorylated S6 or Pdx1 expression in female mTORKO^pl^ fetuses (ESM Fig. 6d, e), which bolsters our finding that increased BCAAs are not sufficient to increase beta cell proliferation in our mTORKO^pl^ offspring. Culture with SNAT AA medium for 3 days significantly increased proliferation of insulin-positive cells in the embryonic pancreas (Fig. 8j, k).

## Discussion

We have demonstrated that placental mTOR deficiency in vivo increases placental amino acid and glucose transporter systems and leucine flux in late-gestation female fetuses only, despite the presence of FGR in our mouse model. Upregulation of nutrient transporters was paradoxically associated with decreased mitochondria function in the female mTORKO placentas. Female mTORKO^pl^ fetuses displayed significantly reduced circulating insulin without perturbations in neonatal islet insulin secretion. Average beta cell size and proliferation were elevated in female mTORKO^pl^ fetuses, which may contribute to increased susceptibility to type 2 diabetes. Collectively, our study reveals a vital role for mTOR signalling in the modulation of placental function, the fetal nutrient environment and early-life offspring beta cell programming.

Mitochondrial function is important for placental development and optimal function. Previous studies have shown that mTORC1 reduction in primary human trophoblasts leads to defects in mitochondrial biogenesis [10]. Our findings of complex I-specific dysfunction, reduced ATP production, electron-transparent mitochondria and disorganised ultrastructure morphology in the female mTORKO placenta align with a poorly functioning placenta. The presence of electron-transparent mitochondria may indicate electron leakage, which may create reactive oxygen species and accelerate cell ageing and damage [30]. One study reported that electron leaks in smooth muscle cells are the result of complex I dysfunction specifically contributing to production of reactive oxygen species [36]. Mitochondrial dysfunction and reactive oxygen species–antioxidant pathways increase glycogen cell accumulation and triacylglycerol nutrient stores in the placenta [37], as seen in the mTORKO placenta. Little is known about mTOR regulation of glycogen cell accumulation and migration. Due to unchanged glucose transport in our model, we speculate that there is an mTOR-mediated delay in glycogen cell migration into the maternal decidua.

Increased amino acid transporter levels and flux in the mTOR-deficient placenta are contrary to many previous reports [7, 11, 13, 38], which made use of primary human trophoblasts and placental villous explants post-birth, and thus may not recapitulate the intrauterine environment. Human studies of FGR report reduced transplacental leucine flux [39]. However, mTOR status in these samples is unknown. Our current study is the first to assess nutrient transport in placentas with mTOR deficiency in vivo and to determine direct consequences on the developing metabolic tissues in the fetus.

Leucine treatment impairs beta cell progenitor proliferation and development when given during pancreatic bud maturation [34]. Reports of leucine lowering insulin expression [34] and increasing proliferation in Pdx1+ progenitor cells [35] suggest its role in beta cell development. These studies implicate mTOR signalling in leucine-mediated proliferation. Beta cell mTOR signalling is a key regulator of cell growth and insulin secretion [40, 41], and governs beta cell nutrient sensing transition during the neonatal period [41]. Despite increased leucine flux in our model, beta cell mTOR signalling was not altered, thus, in these cells, mTOR nutrient sensing does not account for the increased beta cell size or proliferation in female mTORKO^pl^ fetuses.

The impact of SNAT AAs on beta cell proliferation is less well understood. Glutamine amplifies insulin secretion [42] and indirectly enhances beta cell proliferation in primary adult islets [43], but has an unknown impact during development. Incubation of pancreatic bud explants with alanine does not affect proliferation [34], but the effect of alanine in late gestation is unstudied. Incubation of postnatal day 28 beta cells with serine significantly increased beta cell proliferation [44], but the consequences of increased fetal serine on beta cell proliferation are unknown. In our study, fetal pancreas explants were treated with a combination of SNAT AAs to test their capacity to induce proliferation. We show for the first time that SNAT AAs are sufficient to increase beta cell proliferation during late gestation and may negatively impact beta cell development.

Despite increased beta cell size and proliferation in female mTORKO^pl^ fetuses, circulating insulin was lower in late gestation. An early increase in beta cell proliferation in response to nutrient stress can indicate immaturity [45, 46] and lead to early exhaustion [47, 48], which may be underlying long-term beta cell adaptation defects in mTORKO^pl^ female offspring. Additionally, decreased fetal insulin may partially contribute to the FGR phenotype in our model due to its role in promoting growth. We have demonstrated that placental mTOR deficiency is sufficient to induce FGR. However, our data suggest a compensation in amino acid transport in late gestation that has not been reported in humans. It is unlikely that direct deletion of placental mTOR physiologically recapitulates the complexity of human FGR. One limitation of our study is the non-statistically significant decrease in mTOR expression in the male mTORKO placenta. As such, the distinct phenotypes observed in mice of each sex during the neonatal period and adulthood [8] warrant further investigation and substantiation in humans. Understanding the determinants of long-term metabolic health will provide a more accurate picture of how biology and environment interact to influence fetal programming and metabolic health.

## Supplementary Information

Below is the link to the electronic supplementary material.

ESM (PDF 3339 KB)

## Data Availability

Data are available on request from the corresponding author.

## Abbreviations

AGA: Appropriate-for-gestational age
BCAA: Branched chain amino acid
FGR: Fetal growth restriction
GSIS: Glucose-stimulated insulin secretion
HG: High glucose
LG: Low glucose
LSIS: Leucine-stimulated insulin secretion
mtDNA: Mitochondrial DNA
mTOR: Mechanistic target of rapamycin
mTORKO: mTOR knockout
mTORKO^pl^: Offspring exposed to mTOR knockout placenta
SNAT AA: System A amino acid
