# Supplementary material for: Placental mTOR signalling links mitochondrial dysfunction, nutrient transport and neonatal beta cell perturbations in mice
Source: Diabetologia. 2025 Oct 9;68(12):2823–39. doi: 10.1007/s00125-025-06542-z (PMC12594745; doi:10.1007/s00125-025-06542-z)
Supplement: Supplementary file 1 — ESM (PDF 3339 KB) [file 125_2025_6542_MOESM1_ESM.pdf]

Electronic Supplementary Materials – Methods Details

Mouse model and placenta genotyping

Cyp19-cre mice were generated and gifted by Dr. Gustavo Leone (Medical College of Wisconsin). The loxP-flanked mTOR gene was deleted using the Cyp19-cre using a breeding scheme whereby floxed mTOR male mice (stock no. 011009) were crossed with floxed mTOR female mice expressing Cyp19. Due to the mixture of cell types present in the placenta, our model does not display complete deletion of mTOR in placenta lysates measured. To enrich for trophoblasts using FACS in our RNA sequencing study, CAG-GFP was bred into the Cyp19-cre; mTOR floxed line and compared to Cyp19-cre; CAG. All mice were group-housed under 14:10 light-dark cycle with ad libitum access to standard chow. Placental genotypes were determined using standard PCR genotyping on offspring tail tissue.

Cyp19-cre	Forward	GAC CTT GCT GAG ATT AGA TC
	Reverse	GAC GAT GAA GCA TGT TTA GCT GGC C
mTOR flox	Forward	TTT AGG ACT CCT TCT GTG ACA TAC ATT TCC T
	Reverse	TTA TGT TTG ATA ATT GCA GTT TTG GCT AGC AGT
SRY	Forward	TTG TCT AGA GAG CAT GGA GGG CCA TGT CAA
	Reverse	CCA CTC CTC TGT GAC ACT TTA GCC CTC CGA
CAG	WT Fwd	AAG GGA GCT GCA GTG GAG TA
	WT Rev	CCG AAA ATC TGT GGG AAG TC
	Mut Fwd	AAC CAG AAG TGG CAC CTG AC
	Mut Rev	GGC ATT AAA GCA GCG TAT CC

BeWo culture conditions

Cells were cultured in 50% F12K (ATCC, #30-2004) and 50% DMEM (high glucose, +L-glutamine) supplemented with 10% FBS, 1% penstrep, 1% sodium pyruvate with a 3mM final concentration L-glutamine in 37°C, 5% CO^2 incubator.

Human placenta collection and processing

5 g of sample was collected from the mid-section of the placenta. Three biopsies were taken randomly from the body of the placenta and one sample taken near the umbilical cord. Four

1 cm^3 sections from each of the four quadrants of the placenta were washed in PBS and snap frozen within 30 minutes of placental separation at delivery.

Bulk RNA sequencing and gene set enrichment analysis (GSEA)

Bulk RNA sequencing was performed using GFP-sorted trophoblast cells isolated from mouse placentae. Quality control, data alignment, and gene quantification were analyzed using the CHURP pipeline (Baller et al., 2019) at the University of Minnesota Supercomputing Institute (MSI). 2 x 150bp FASTQ paired-end reads for 16 samples (74.7 million reads average per sample) were trimmed using Trimmomatic (v0.33) enabled with the optional "headcrop -3" option, "-q" option; 3bp sliding-window trimming from 3' end requiring minimum Q30. Quality control on raw sequence data for each sample was performed with FastQC. Read mapping was performed via HISAT2 (Kim et al., 2019) (v2.1.0) using the mouse genome (GRCm39.109) as a reference. Gene quantification was done via Feature Counts for raw read counts. Differentially expressed genes were identified using the edgeR (negative binomial, R programming) feature in CHURP using raw read counts. We filtered the generated list based on a minimum log2 fold change > [1] and FDR corrected $p < 0.05$. For GSEA analysis, GSEA was performed following www.gsea-msigdb.org/gsea/index.jsp, using the Hallmark gene sets, which represent well-defined biological states and processes. GSEA reference: Subramanian A, Tamayo P, Mootha VK et al. (2006) Gene set enrichment analysis: a knowledge-based approach for interpreting genome-wide expression profiles. Proc Natl Acad Sci USA 102(43):15545-15550. https://doi.org/10.1073/pnas.0506580102. Mootha VK, Lindgren CM, Eriksson K-F et al. (2003) PGC-1-responsive genes involved in oxidative phosphorylation are coordinately downregulated in human diabetes. Nat Genet 34(3):267-273. https://doi.org/10.1038/ng1180, Baller, J., Kono, T., Herman, A., and Zhang, Y. CHURP: A Lightweight CLI Framework to Enable Novice Users to Analyze Sequencing Datasets in Parallel. In Practice and Experience in Advanced Research Computing 2019: Rise of the Machines (learning) (PEARC '19). Association for Computing

Machinery, New York, NY, USA, Article 96, 1–5 (2019). https://doi.org/10.1145/3332186.3333156,

Kim, D., Paggi, J.M., Park, C. et al. Graph-based genome alignment and genotyping with HISAT2 and HISAT-genotype. Nat Biotechnol 37, 907–915 (2019). https://doi.org/10.1038/s41587-019-0201-4

Western blotting antibodies

mTOR (Cell Signaling Technologies (CST)), 2983S, 1:500), SNAT1 (CST, 36057S; 1:500), total OxPhos (Abcam, ab110413; 1:1000), vinculin (CST, 13901S; 1:1000).

Frozen placenta sample and drug preparation for Seahorse

Flash frozen mouse placentae (e17.5) were homogenized with MAS buffer, centrifuged for 10 minutes at 1000xg at 4°C, and pellet was discarded. Following protein quantification by BCA assay, 80 ug sample was plated in Seahorse XF96 microplate. The plate was centrifuged for 5 minutes at 2000xg at 4°C. Cytochrome C (equine heart) prepared in MAS buffer was loaded into each well containing sample. Seahorse Flux plate was prepared with the following injections: A (NADH or succinate), B (rotenone/antimycin A), C (TMPD/ascorbic acid), and D (azide).

Immunohistochemistry analysis

For mouse placenta, images were taken at 4x and 60x magnification. Analysis of protein expression by chromogen immunostaining intensity was performed on the 4x images using ImageJ software as previously described (27). Analysis was performed for the whole placenta and by zone (manually determined). Fold-change was used to normalize to respective male or female controls. For human placenta, 3 images were randomly captured at 20x magnification and chromogen immunostaining intensity analysis was performed. Browning intensity was calculated and divided by the number of nuclei per image.

Beta cell and alpha cell mass analysis

FIJI ImageJ software was used to quantify hormone-positive area across pancreas sections. The values of the 5-6 tissues per animal were averaged and the insulin-positive to total pancreas ratio was multiplied by the pancreas weight of the animal to obtain its beta cell mass. Similarly, the glucagon-positive to total pancreas ratio was multiplied by the pancreas weight of the animal to obtain its alpha cell mass. Average beta cell size was obtained by dividing the overall beta cell area by the number of beta cells in each islet image quantified. Beta cell-specific Ki67 percentages were obtained by counting the Ki67-positive beta cells, dividing that number by the DAPI positive beta cells and multiplying the resulting value by 100. Analysis of beta cell-specific Ki67 was performed on 2-3 sections per pancreas and averaged. Beta cell-specific phosphorylated ribosomal S6 and pancreas-wide Pdx1 staining were analyzed using Fiji ImageJ selection tool to manually select area to measure mean and area. The mean was normalized to the area and averaged per animal.

Dynamic glucose- and leucine-stimulated insulin secretion

For static GSIS, 10 size-matched islets were equilibrated at 37°C in Krebs Buffer with 2 mM glucose (LG) for 2 hours. Islets tested in triplicate were incubated in LG for 30 minutes before collection of the supernatant and transfer into sequential incubations of 16.7 mM high glucose (HG, 30 min), LG rest (15 min, discarded), and 30 mM KCl (15 min). Mesh screens containing islets were collected into 500 μL lysis buffer (10% RIPA + 1% protease inhibitor cocktail).

Dynamic GSIS perifusion experiments were performed using a PERI4-V2 machine (Biorep Technologies, Miami, FL, USA). Batches of 40-60 islets were handpicked and loaded into perifusion chambers. Perifusion buffer containing sequential concentrations of glucose (2 mM, 16.7mM) and glucose plus leucine (1 mM) was circulated through the columns and collected in 96-well plates. For each run, a 45-60 minute incubation of low glucose (2 mM) washing at a rate of 100uL/min served to stabilize islets before stimulation in the following sequence: 16 minutes of

low glucose (2 mM), 16 minutes of low glucose (2 mM) plus leucine (1 mM), 24 minutes of high glucose (16.7 mM), 24 minutes of high glucose (16.7 mM) plus leucine (1 mM), 8 minutes of low glucose (2 mM) rest, and 8 minutes of KCl (30 mM). Samples (100uL) were collected every minute from the outflow tubing into a 96-well plate. Islets and perifusion solutions were kept at 37ºC in a temperature-controlled chamber while the perfusate was kept at 4ºC. Islets within perifusion chambers were recovered, washed with HBSS, and stored in -80ºC until lysis. Any timepoint with below the threshold of detection in insulin ELISA were substituted as 0.

Embryonic pancreas explant culture

RPMI 1640 media was supplemented with 5 mM glucose, 10% FBS, pen/strep, and the following amino acids: 1 mM leucine, 1 mM isoleucine, 1 mM valine (BCAAs) or 4 mM glutamine, 0.1 mM alanine, 0.6 mM serine (SNAT AAs). Amino acid concentrations are approximately 2-3x higher than standard RPMI 1640 media used as control.

ESM TABLE 1

		AGA	FGR	p-value
**Maternal age at delivery**		32.40 ± 5.549	29.75 ± 10.436	p=0.6372
**Maternal ethnicity**	Caucasian	4	1	
	African American	0	2	
	Asian	1	0	
	Other	0	1	
**Mode of delivery**	Vaginal	0	1	
	Cesarean	5	3	
**Gestational age**		38.48 ± 1.089	38.6 ± 1.395	p=0.8884
**Fetal sex**	Male	3	0	
	Female	2	4	
**Birth weight**		3409.2 ± 197.99	2376.5 ± 186.43	p=9.3256E-05
**Placental weight**		597.42 ± 106.12	442.45 ± 73.93	p=0.04298

ESM TABLE 2

Gene	Forward primer	Reverse primer
Slc38a1 (SNAT1)	GGCATCTGTATTTGCTGCTG	CGTTGCTGACGTTGTCATCT
Slc38a2 (SNAT2)	TAATCTGAGCAATGCGATTGTGG	AGATGGACGGAGTATAGCGAAAA
Slc38a4 (SNAT4)	GCGGGGACAGTATTCAGGAC	GGAACTTCTGACTTTCGGCAT
Slc7a5 (LAT1)	GATCATCTCCTCCGTGACAAA	TCCTTTGAAGGCACCAATCT
Slc7a8 (LAT2)	TCAGCGCCTGTGGTATCATTG	TGATGCCTGTCACGATCCAGA
Slc43a2 (LAT4)	GCTGATTGCATATGGAGCAAGTAAC	CGAAGTGAACGTCATGCACAT
Slc2a1 (GLUT1)	GATCACTGCAGTTCGGCTATAA	CGGTGGTTCCATGTTTGATTG
Slc2a2 (GLUT2)	ACACCGGAATGTTCTTAGCC	GTGAGAAGCCGAGGAAAG
Slc2a3 (GLUT3)	CACTGGATCCCACTGTCACC	TAGAGGAGTCGCCTTTCCCA
Slc2a4 (GLUT4)	AGCGAGTGACTGGAACACTG	TTGTGGGATGGAATCCGGTC
Drp1	ATGCCAGCAAGTCCACAGAA	TGTTCTCGGGCAGACAGTTT
Opa1	AAGTGGATTGTGCCTGACTTT	CAACCCGTGGTAGGTGATCT
Mfn1	TTGCCACAAGCTGTGTTCGG	TCTAGGGACCTGAAAGATGGGC
Mfn2	AGAGGCAGTTTGAGGAGTGC	ATGATGAGACGAACGGCCTC
Tfam	TCCACAGAACAGCTACCCAA	CCACAGGGCTGCAATTTTCC
Nrf1	AGAAACGGAAACGGCCTCAT	CATCCAACGTGGCTCTGAGT
Nrf2	ATGGAGCAAGTTTGGCAGGA	GCTGGGAACAGCGGTAGTAT
Pparg	TGTGGGGATAAAGCATCAGGC	CCGGCAGTTAAGATCACACCTAT
Pparc1a	TCAAGCCAAACCAACAACTTTATCT	GGTTCGCTCAATAGTCTTGTTCTCA
Beta actin	GCC CTG AGG CTC TTT TCC AG	TGC CAC AGG ATT CCA TAC CC

ESM TABLE 3

IF staining – e17.5 pancreas			
**Target**	**Catalog number**	**Primary antibody concentration**	**Secondary antibody**
Insulin (Guinea pig)	Sigma I8510	1:10,000	1:400 (Anti-Guineapig FITC, Jackson Immunoresearch #106-005-003)
Glucagon (Rabbit)	Abcam ab92517	1:10,000	1:400 (Anti-Rabbit Cy3, Jackson Immunoresearch #111-005-144)
Ki67 (Rabbit)	ThermoFisher	1:1000	1:400 (Anti-Rabbit Cy3, Jackson Immunoresearch #111-005-144)
Phospho-S6 S240/244 (Rabbit)	Cell Signaling Technology 5364S	1:1000	1:400 (Anti-Rabbit Cy3, Jackson Immunoresearch #111-005-144)
Pdx1 (Goat)	Abcam ab47383	1:1000	1:400 (Donkey Anti-Goat Cy3, Jackson Immunoresearch #705-165-147)
IHC staining – e17.5 placenta			
**Target**	**Catalog number**	**Primary antibody concentration**	**Secondary antibody**
LAT1 (Rabbit)	Proteintech 13752-1-AP	1:500	Rabbit specific HRP/DAB (ABC) Detection IHC Kit Catalog number: ab64261
LAT4 (Rabbit)	Invitrogen PA5-23571	1:500	
SNAT1 (Rabbit)	Cell Signaling Technology 36057S	1:500	

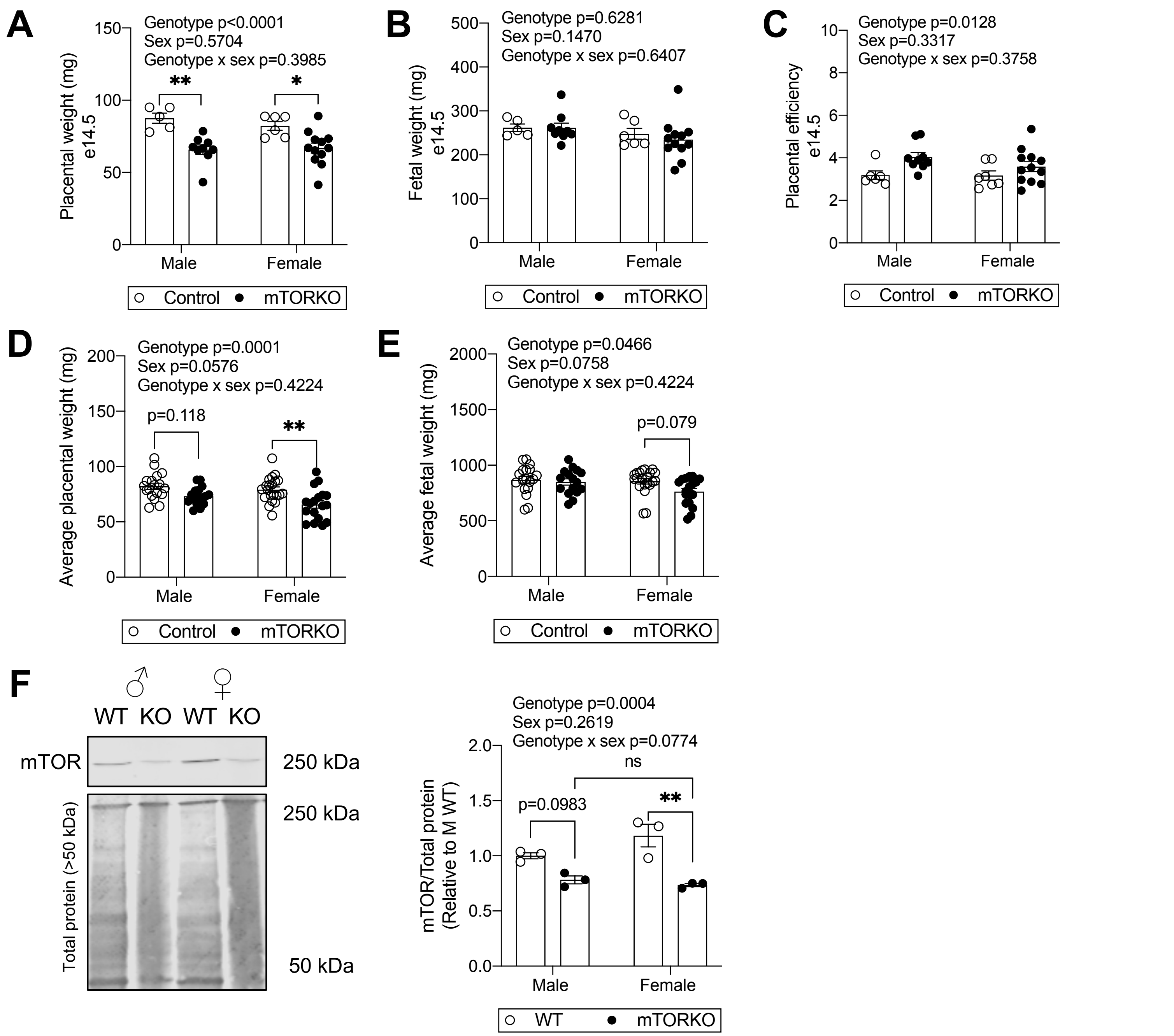

ESM Figure 1. Placental mTOR deficiency reduces placental weight but not fetal weight or placental efficiency during mid-gestation. (A) Weights of control and mTORKO male and female e14.5 placentae (n=5-12). (B) Weights of control and mTORKO^{pl} male and female e14.5 fetuses (n=5-12). (C) Placental efficiency of control and mTORKO^{pl} male and female e14.5 offspring determined by fetal weight divided by placental weight (n=5-12). (D) Average weight per litter of control and mTORKO male and female e17.5 placentae (n=15-20 litters). (E) Average weight per litter of control and mTORKO^{pl} male and female e17.5 fetuses (n=15-20 litters). (F) Western blot and mTOR protein quantification of wildtype and mTORKO placentae (n=3). Statistical analyses were conducted using two-way ANOVA with sex and genotype as factors with Tukey's post hoc test and significance *p<0.05, **p<0.01.

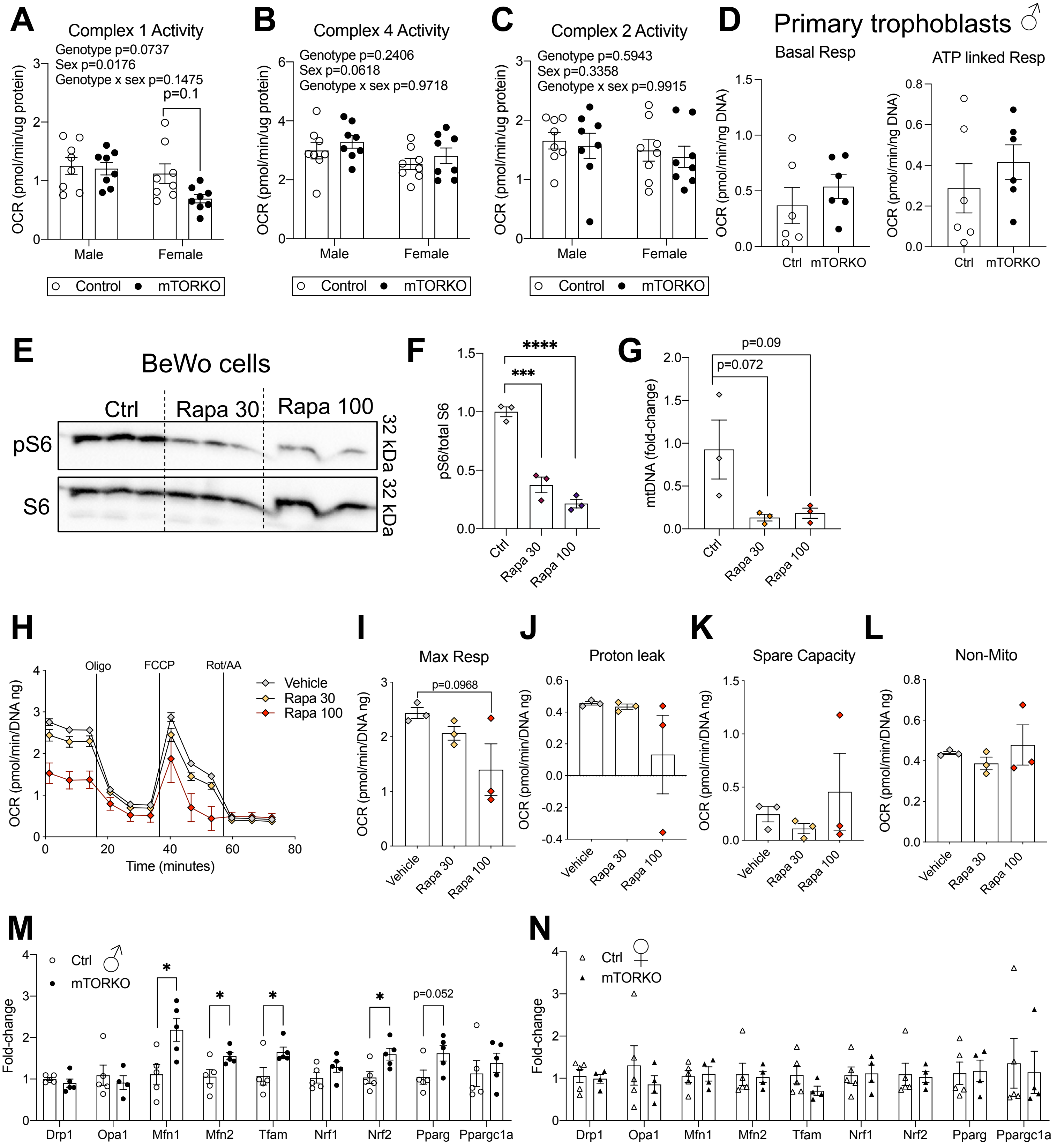
A
Complex 1 Activity
Genotype p=0.0737
Sex p=0.0176
Genotype x sex p=0.1475
p=0.1
OCR (pmol/min/ug protein)
Male
Female
Control
mTORKO
B
Complex 4 Activity
Genotype p=0.2406
Sex p=0.0618
Genotype x sex p=0.9718
OCR (pmol/min/ug protein)
Male
Female
Control
mTORKO
C
Complex 2 Activity
Genotype p=0.5943
Sex p=0.3358
Genotype x sex p=0.9915
OCR (pmol/min/ug protein)
Male
Female
Control
mTORKO
D
Primary trophoblasts ♂
Basal Resp
ATP linked Resp
OCR (pmol/min/ng DNA)
Ctrl
mTORKO
E
BeWo cells
Ctrl
Rapa 30
Rapa 100
pS6
S6
32 kDa
32 kDa
F
pS6/total S6
****
***
Ctrl
Rapa 30
Rapa 100
G
mtDNA (fold-change)
p=0.09
p=0.072
Ctrl
Rapa 30
Rapa 100
H
Oligo
FCCP
Rot/AA
Vehicle
Rapa 30
Rapa 100
OCR (pmol/min/DNA ng)
Time (minutes)
I
Max Resp
p=0.0968
J
Proton leak
K
Spare Capacity
L
Non-Mito
OCR (pmol/min/DNA ng)
Vehicle
Rapa 30
Rapa 100
M
Ctrl ♂
mTORKO
Fold-change
*
*
*
*
p=0.052
Drp1
Opa1
Mfn1
Mfn2
Tfam
Nrf1
Nrf2
Pparg
Ppargc1a
N
Ctrl ♀
mTORKO
Fold-change
Drp1
Opa1
Mfn1
Mfn2
Tfam
Nrf1
Nrf2
Pparg
Ppargc1a

ESM Figure 2. Mitochondrial function and gene expression in mouse primary trophoblasts and BeWo cells treated with rapamycin. Quantification of (A) complex 1, (B) complex 4, and (C) complex 2 activity analyzed by two-way ANOVA. (D) Basal respiration and ATP-linked respiration in primary trophoblasts isolated from male control and mTORKO placentae (n=6). (E) Western blot image measuring phosphorylated S6 (pS6) and total S6 (S6) in BeWo cells treated with 30 nm or 100 nM rapamycin for 24 hours. (F) Quantification of pS6/S6 (n=3). (G) Mitochondria mass quantified by mtDNA in control and rapamycin-treated BeWo cells. (n=3). (H) Seahorse trace and quantification of (I) maximal respiration, (J) protein leak, (K) spare capacity, and (L) non-mitochondrial respiration in rapamycin-treated BeWo cells using Seahorse (n=3). Gene expression of mitochondria-related genes in control and mTORKO (M) male and (N) female placentae (n=4-6). Statistical analyses were conducted using an unpaired two-tailed t-test or one-way ANOVA with Tukey’s post hoc test and significance *$p<0.05$, ***$p<0.001$, ****$p<0.0001$.

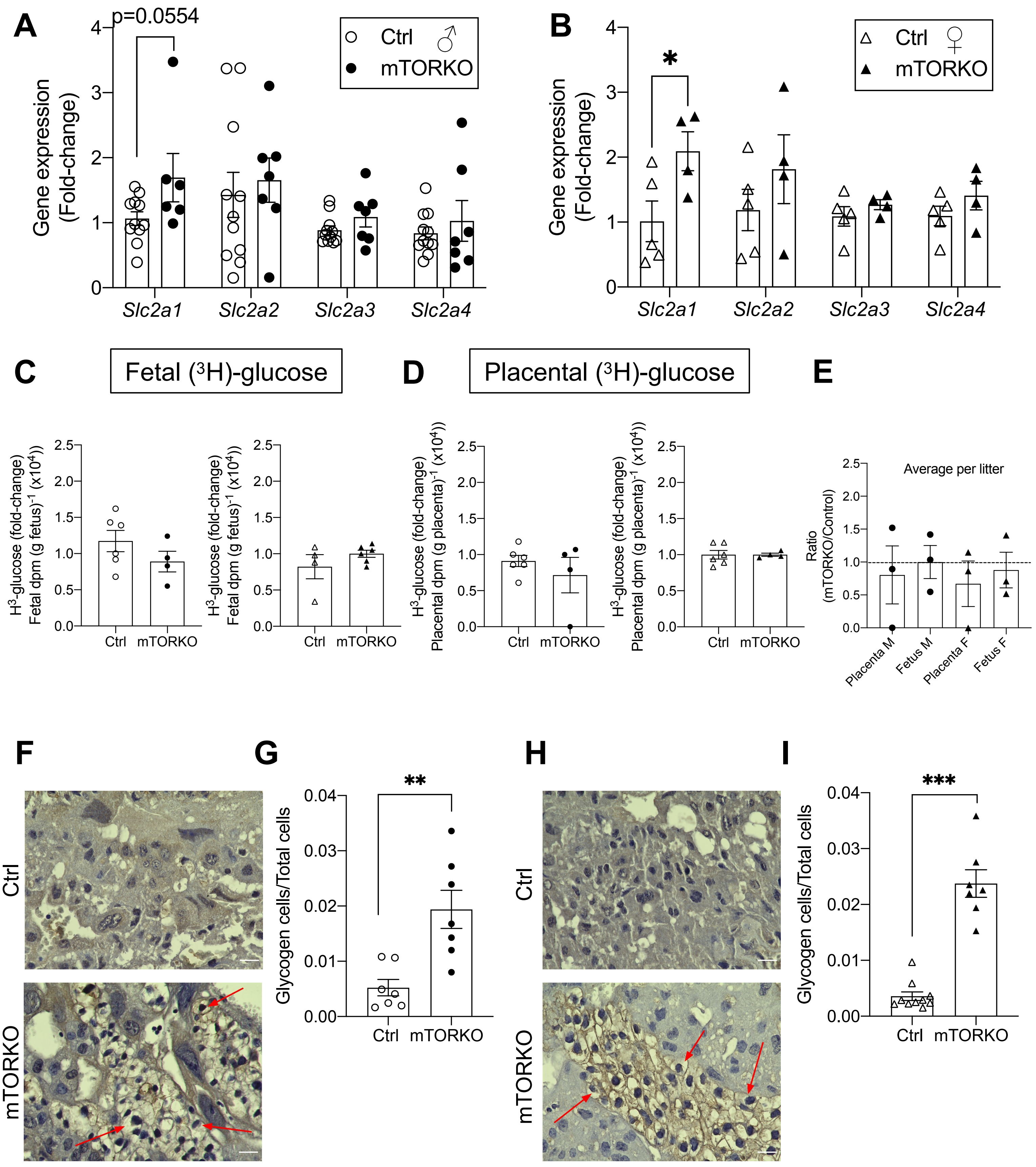
A
p=0.0554
Ctrl ♂
mTORKO
Gene expression (Fold-change)
Slc2a1
Slc2a2
Slc2a3
Slc2a4
B
*
Ctrl ♀
mTORKO
Gene expression (Fold-change)
Slc2a1
Slc2a2
Slc2a3
Slc2a4
C
Fetal (3H)-glucose
H3-glucose (fold-change) Fetal dpm (g fetus)-1 (x104))
Ctrl
mTORKO
D
Placental (3H)-glucose
H3-glucose (fold-change) Placental dpm (g placenta)-1 (x104))
Ctrl
mTORKO
E
Average per litter
Ratio (mTORKO/Control)
Placenta M
Fetus M
Placenta F
Fetus F
F
Ctrl
mTORKO
G
**
Glycogen cells/Total cells
Ctrl
mTORKO
H
Ctrl
mTORKO
I
***
Glycogen cells/Total cells
Ctrl
mTORKO

ESM Figure 3. Glucose transport expression, glucose flux, and glycogen cells in late-gestation. *Slc2a1* (GLUT1), *Slc2a2* (GLUT2), *Slc2a3* (GLUT3), and *Slc2a4* (GLUT4) gene expression in (A) male and (B) female control and mTORKO placentae (n=4-11). (C) Tritium-labeled glucose detected in male and female fetuses following administration to maternal circulation (n-5-6). (D) Tritium-labeled glucose detected in male and female placentae following administration to maternal circulation (n-5-6). (E) Ratio of H^{3-}glucose counts in mTORKO/Control averaged per litter (n=3). Representative images and quantification of glycogen cells in the junctional zone of (F-G) male control and mTORKO (n=7) and (H-I) female control and mTORKO placentae (n=7-10). Statistical analyses were conducted using an unpaired two-tailed t-test with significance *p<0.05, **p<0.01, ****p<0.0001.

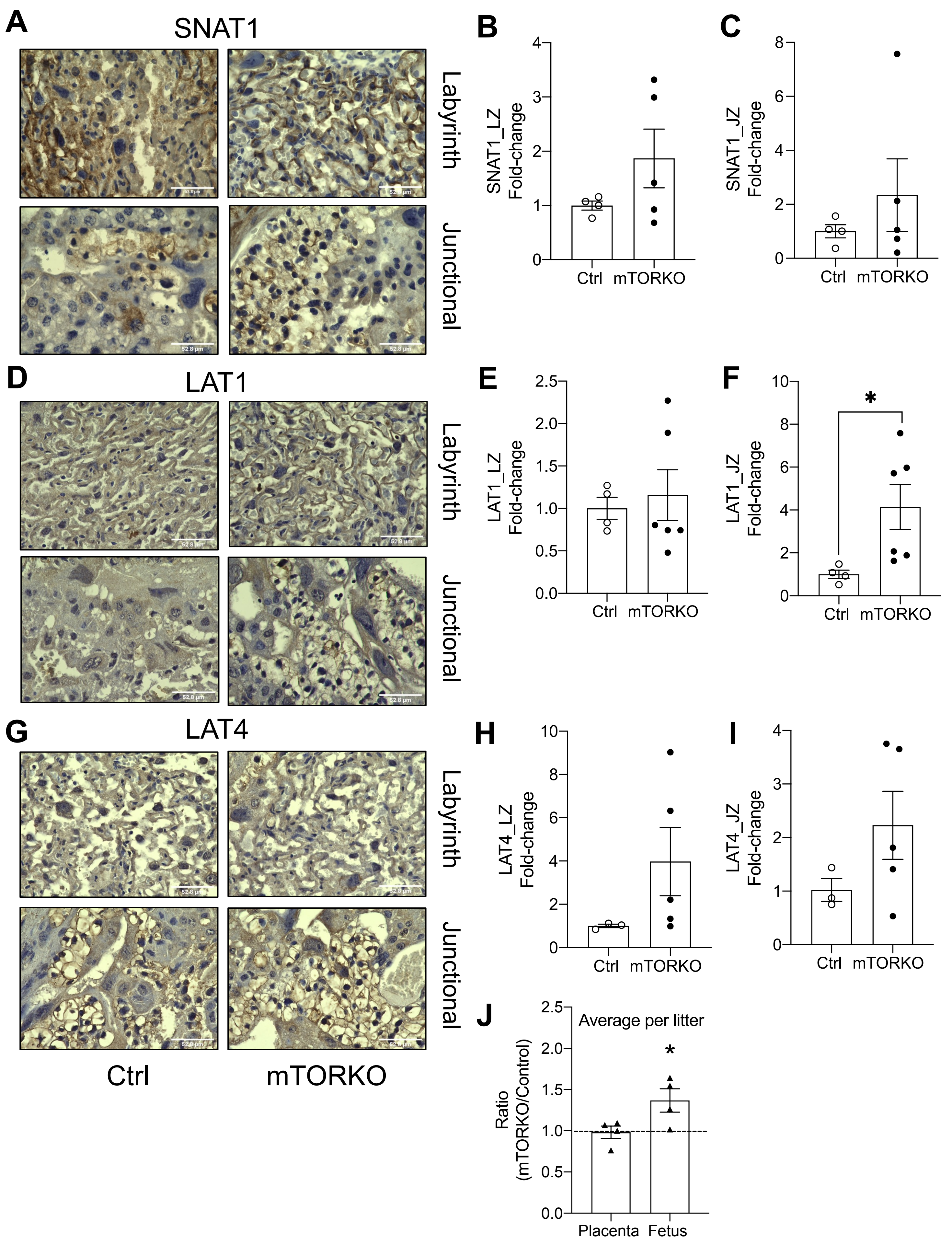

A
SNAT1
Labyrinth
Junctional
B
SNAT1_LZ Fold-change
Ctrl
mTORKO
C
SNAT1_JZ Fold-change
Ctrl
mTORKO
D
LAT1
Labyrinth
Junctional
E
LAT1_LZ Fold-change
Ctrl
mTORKO
F
*
LAT1_JZ Fold-change
Ctrl
mTORKO
G
LAT4
Labyrinth
Junctional
Ctrl
mTORKO
H
LAT4_LZ Fold-change
Ctrl
mTORKO
I
LAT4_JZ Fold-change
Ctrl
mTORKO
J
Average per litter
*
Ratio (mTORKO/Control)
Placenta
Fetus

ESM Figure 4. System A and system L amino acid transporter expression in the male mTORKO placenta. (A) 60X images of SNAT1 IHC staining by zone in male control and mTORKO placentae. Quantification of (B) labyrinth zone and (C) junctional zone (n=4-5); normalized to male control placenta by fold-change. (D) 60X images of LAT1 IHC staining by zone in male control and mTORKO placentae. Quantification of (E) labyrinth zone and (F) junctional zone (n=4-6); normalized to male control placenta by fold-change. (G) 60X images of LAT4 IHC staining by zone in male control and mTORKO placentae. Quantification of (B) labyrinth zone and (C) junctional zone (n=4-5); normalized to male control placenta by fold-change. (J) Ratio of H^3-leucine counts in mTORKO/Control averaged per litter (n=4). Statistical analyses were conducted using an unpaired two-tailed t-test with significance *$p<0.05$.

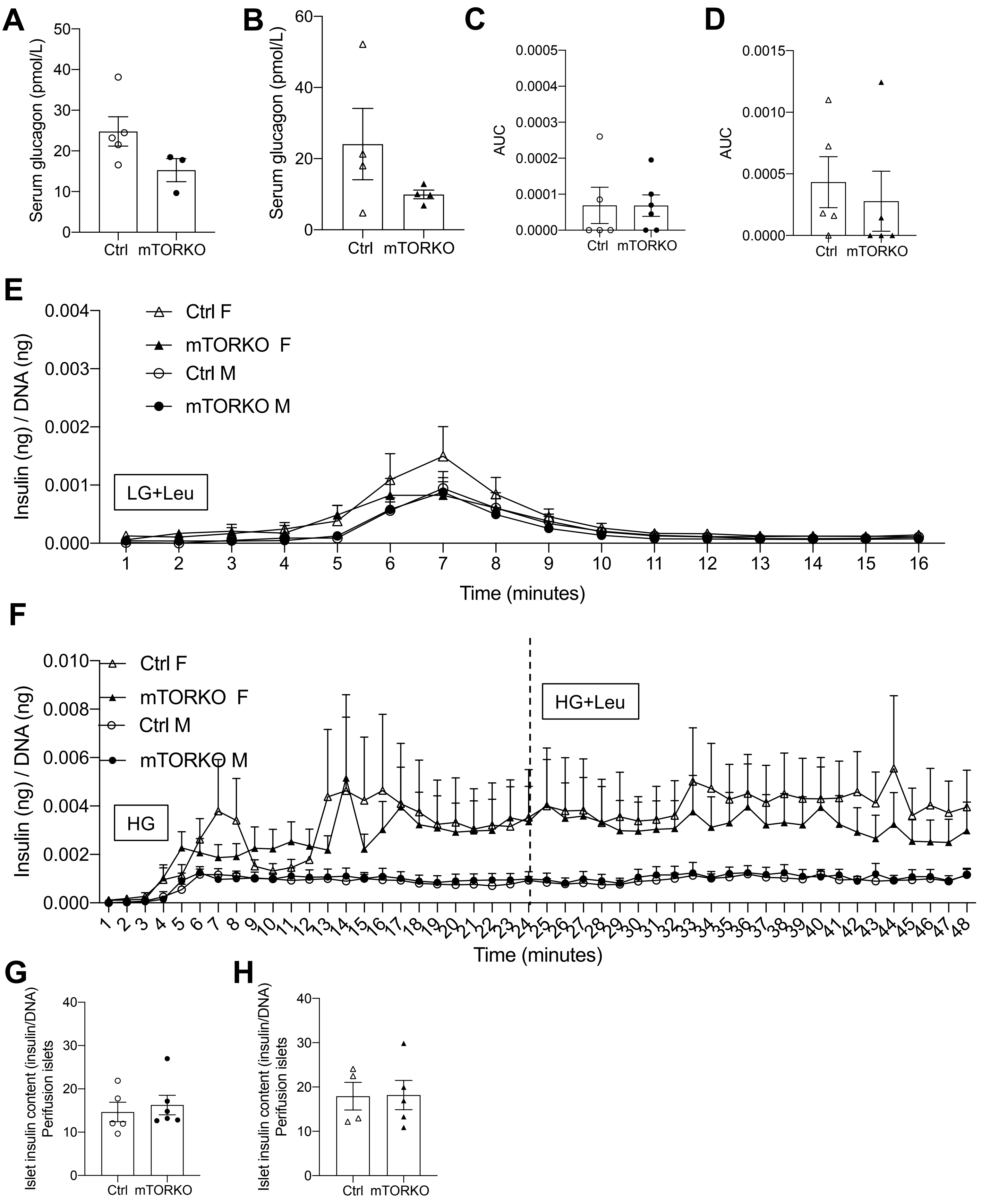

A
Serum glucagon (pmol/L)
Ctrl
mTORKO
B
Serum glucagon (pmol/L)
Ctrl
mTORKO
C
AUC
Ctrl
mTORKO
D
AUC
Ctrl
mTORKO
E
Ctrl F
mTORKO F
Ctrl M
mTORKO M
LG+Leu
Insulin (ng) / DNA (ng)
Time (minutes)
F
Ctrl F
mTORKO F
Ctrl M
mTORKO M
HG
HG+Leu
Insulin (ng) / DNA (ng)
Time (minutes)
G
Islet insulin content (insulin/DNA)
Perifusion islets
Ctrl
mTORKO
H
Islet insulin content (insulin/DNA)
Perifusion islets
Ctrl
mTORKO

ESM Figure 5. Serum glucagon and GSIS traces of male and female mTORKO^{pl} neonatal islets. (A) Serum glucagon levels in late-gestation (e17.5) control and mTORKO^{pl} (A) male and (B) female fetuses (n=3-5). (C-D) Dynamic GSIS response to low glucose (LG, 2mM) condition in control and mTORKO^{pl} male (n=5-6) and female (n=4-5) neonates presented as area under curve (AUC). (E) Dynamic GSIS traces across 16-minute low glucose (LG, 2 mM) + leucine (1 mM, LG+leu) incubation of male and female control and mTORKO^{pl} neonatal islets. Zero timepoint denotes start of LG+leu perfusion. (F) Dynamic GSIS traces across 24-minute high glucose (HG, 16.7 mM) and 24-minute HG + leucine (1 mM, HG+leu) incubation of male and female control and mTORKO^{pl} neonatal islets. Zero timepoint denotes start of HG perfusion. Islet insulin content of (G) male and (H) female control and mTORKO^{pl} neonates (n=4-6). Statistical analyses were conducted using an unpaired two-tailed t-test with significance $^*p<0.05$.

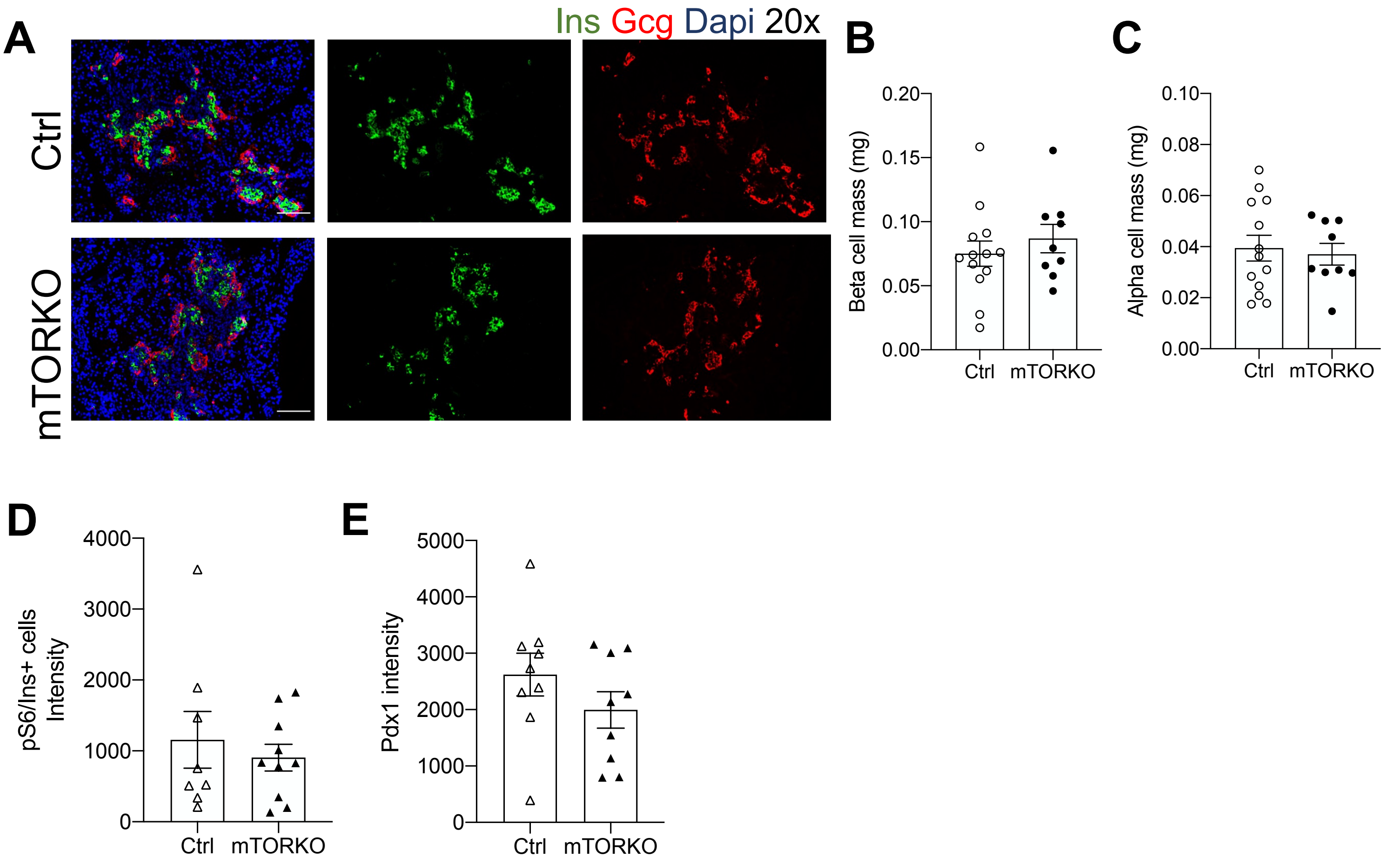

ESM Figure 6. Endocrine cell mass in male fetuses exposed to placenta mTOR deficiency. (A) 20X representative images of islets stained for insulin (green) and glucagon (red) from male littermate control and mTORKO^{pl} fetuses. Scale bar = μm. Quantification of (B) Beta cell mass and (C) alpha cell mass (n=9-13). (D) Quantification of mTOR signaling in insulin-positive cells determined by phosphorylated S6 in islets from female control and mTORKO^{pl} fetuses. (DPdx1 intensity in pancreas from female control and mTORKO^{pl} fetuses. Statistical analyses were conducted using an unpaired two-tailed t-test with significance *p<0.05.
